# Maternal Near-Miss as a Surrogate Indicator of the Quality of Obstetric Care: A Study in a Tertiary Care Hospital in Eastern India

**DOI:** 10.7759/cureus.12548

**Published:** 2021-01-07

**Authors:** Vinita Singh, Archana Barik

**Affiliations:** 1 Obstetrics and Gynaecology, Tata Main Hospital, Jamshedpur, IND

**Keywords:** maternal near-miss, maternal death, obstetric care

## Abstract

Background

Monitoring severe acute maternal morbidity or maternal near-miss is currently proposed by WHO as a valuable tool to assess the quality of obstetric care and implement new strategies for improving maternal health.

Aim and objective

The objective of this study was to assess and analyze the incidence of maternal near-miss (MNM) and maternal death (MD) at Tata Main Hospital, Jamshedpur, a tertiary care hospital in eastern India.

Material and method

This study was a prospective observational study conducted at Tata Main Hospital from November 2016 to October 2019. The study population included all the pregnant women who fulfilled the WHO near-miss criteria based on organ dysfunction or failure and all the maternal deaths that occurred during the study period.

Results

During the study period, there were 15,377 deliveries and 14,636 live births. The MNM cases were 153, and 38 were maternal deaths. The maternal near-miss ratio (MNMR) and severe maternal outcome ratio (SMOR) were 19.9 and 13.1 per 1000 live births, respectively. The maternal near-miss to mortality ratio (MNM: 1 MD) was 4:1, and the mortality index (MI) was 19.9%. Haemorrhagic disorders were the leading cause (40.5%) of MNM, followed by hypertensive disorders (25.5%) and cardiac diseases (14.4%). Similarly, both haemorrhage (23.7%) and sepsis (23.7%) were the leading causes of death followed by hypertensive disorders (15.8%). On reviewing patients, 62% of near-miss and 92% of mortality cases had shown organ dysfunction on admission.

Conclusion

MNM and MD cases share similar pathology with a different outcome. Hence, monitoring a larger volume of MNM cases helps in identifying the causes of maternal adverse events and finding out the gaps in the management more effectively than auditing only the maternal deaths.

## Introduction

Reduction in maternal mortality is an essential component of the millennium development goal (MDG) [1]. Despite a significant decline in the number of maternal death, the rate of decline is less than half of what is needed to achieve the MDG target. Resource-limited countries like India and other Asian and African countries take the maximum burden where a woman's lifetime risk of dying during pregnancy and childbirth continues to be high as compared to developed countries.

Nevertheless, maternal deaths are just the tip of the iceberg. The broad base of this iceberg represents many more women who may have nearly died from life-threatening conditions but survived. These are the maternal near-miss cases, defined as a woman who nearly died but survived a complication that occurred during pregnancy, childbirth, or within 42 days of termination of pregnancy [2]. Maternal death is traditionally used by epidemiologists and caregivers as an indicator of maternal health status at all levels starting from a community to the whole country.

However, with continuously declining national figures, the absolute number of maternal mortality in an area or a community may be smaller, and the analysis of a smaller sample size of maternal deaths may not reveal the actual gaps in the health care delivery system. Hence, reviewing the near-miss cases which have a larger sample size can unravel the lacunae more effectively.

Near-miss data is also a tool for policymakers to envisage the requirements of essential and emergency obstetric care, and is helpful in designing, implementing, and following up safe motherhood programs. Therefore, recently many institutions and health care facilities across the world have started reviewing near-miss cases as an effective audit of maternal care.

## Materials and methods

This study was a prospective observational study carried out in the Department of Obstetrics and Gynaecology, Tata Main Hospital, Jamshedpur, a tertiary care hospital in the state of Jharkhand, India. This hospital is a 914-bedded facility with a 32-bedded intensive care unit (ICU), 16-bedded high dependency unit (HDU), and 24-hours blood bank facility. The Obstetrics and Gynaecology (O&G) department has a state-of-the-art 19-bedded labour room that is manned round-the-clock for emergency services. Apart from that, the labour room has five HDU beds that cater to high-risk pregnancies and obstetric emergencies and act as the resuscitation bay. This hospital has a large patient base, and it provides healthcare facilities not only to Jamshedpur but also from other parts of the state.

The study population included all the pregnant women admitted to Tata Main Hospital from November 2016 to October 2019. Based on the clinical audit, the patients who had severe maternal complications, and those who underwent critical interventions or needed ICU admissions (Table 1) were identified. Among them, the patients who fulfil the WHO near-miss criteria based on organ function dysfunction or failure were considered as eligible cases (Table 2). During this period, maternal deaths were also registered.

**Table 1:** Criteria for eligible patients (who had severe maternal complications and or critical interventions)

Severe maternal complications	Critical interventions
Severe postpartum hemorrhage	Admission to the intensive care unit
Severe pre-eclampsia	Interventional radiology
Eclampsia	Laparotomy (includes hysterectomy, excludes cesarean section)
Sepsis or severe systemic infection	Use of blood products (transfusion of blood or red cells (≥ 5 units)
Ruptured uterus	Interventional radiology
Severe complications of abortion	
Ruptured ectopic pregnancy	

**Table 2:** Near-miss criteria based on organ dysfunction or failure PaO_2_- arterial oxygen partial pressure; FiO_2_- fractional inspired oxygen; PT- prothrombin time; aPTT- activated partial thromboplastin time

Organ system dysfunction or failure	Criteria
Cardiovascular dysfunction	Shock
	Cardiac arrest and cardiopulmonary resuscitation
	Use of continuous vasoactive drugs
	Severe hypoperfusion (lactate >5 mmol/l or >45 mg/dl)
	Severe acidosis (pH<7.1)
Respiratory dysfunction	Acute cyanosis
	Gasping
	Respiratory rate >40 or <6,
	Intubation and ventilation (not related to anesthesia)
	Severe hypoxemia (O_2_ saturation <90% for ≥60 minutes or PaO_2_/FiO_2_ < 200)
Renal dysfunction	Oliguria non-responsive to fluids or diuretics
	Dialysis for acute renal failure
	Severe acute azotemia (creatinine ≥3.5 mg/dl)
Coagulation/hematological dysfunction	Severe acute thrombocytopenia (<50 000 platelets/ml)
	PT or aPTT > 1.5 times of normal
Hepatic dysfunction	Jaundice in the presence of pre-eclampsia
	severe acute hyperbilirubinemia (bilirubin >6.0 mg/dl)
Neurological dysfunction	Prolonged unconsciousness (lasting ≥12 hours)
	Coma (including metabolic coma)
	Stroke
	Uncontrollable fits/status epilepticus

The patient characteristics including age, parity, and gestational age at admission were recorded. Antenatal booking status which is defined as more than three antenatal visits to our hospital irrespective of the gestational age was also noted. Apart from that, the condition of the patients during admission, the mode of delivery, the primary cause of morbidity, the sequence of morbidity, organ dysfunction, and the key interventions was recorded. All the cases were followed up during their hospital stay till their discharge or death. The following indicators were calculated; maternal near-miss (MNM), maternal death (MD), severe maternal outcome ratio (SMOR = MNM+ MD per 1000 live births), MNM ratio (MNMR = MNM per 1000 Live births), maternal near-miss: mortality ratio (MNM: 1MD), and mortality index [MI = MD/ (MNM + MD)].

Data collection

All needed data were extracted from the medical records and entered into a study proforma. Individual participants of the study were not interviewed, since no information was directly obtained from the patients. Confidential information about the identity of the individual participants like name, medical registration number, and the date of hospital admission was kept undisclosed by the data collector. As there was no direct interaction with participants, informed consent from the individual patients was not obtained. However, permission from the institutional ethical review committee had been obtained before conducting this study. The data from individual patients were audited and analyzed weekly in a departmental review meeting led by the head of the department (the author of this paper).

## Results

There was a total of 15,377 deliveries and 14,636 live births during the study period. The total number of eligible patients was 345 and out of which 153 patients identified as maternal near-miss cases. There were 38 maternal deaths registered during the study period. The demographic parameters near-miss and mortality cases are shown in Table 3. In the near-miss group, 64% of patients were between the age of 20 to 29 years. In the mortality group, 68.4% of patients were between 25 to 34 years of age. Both morbidity and mortality were higher in multigravida patients. In the near-miss group, 60% of women were more than 28 weeks of gestation on admission, whereas in the mortality group, patients were evenly distributed in the prenatal and postnatal category. Approximately 84% of the near-miss and 92% of the mortality group were un-booked (no prior antenatal visit). 

**Table 3:** Patient characteristics of near-miss cases and maternal deaths

Patients characteristics	Near Miss n (%) (N = 153)	Maternal Death n (%) (N = 38)
Age		
≤19	15 (9.8)	2 (5.3)
20-24	58 (37.9)	10 (26.3)
25-29	40 (26.1)	12 (31.6)
30-34	24 (15.7)	14 (36.8)
≥35	16 (10.5)	0 (0)
Gravida		
Primigravida	57 (37.3)	10 (26.3)
Multigravida	96 (62.7)	28 (73.7)
Gestational Age (Weeks)		
1-12	24 (15.7)	2 (5.3)
13-28	14 (9.2)	0 (0)
>28	92 (60.1)	20 (52.6)
Post Natal	23 (15)	16 (42.1)
Status of antenatal Booking		
Booked	24 (15.7)	3 (7.9)
Un-Booked	129 (84.3)	35 (92.1)

Various quality indicators were calculated year-wise and displayed in Table 4. The average maternal mortality rate (MMR) and maternal near-miss ratio (MNMR) in the study period were 259.6 and 10.5, respectively.

**Table 4:** Year-wise data of maternal near-miss, maternal death, and various quality indicators

Quality Indicators	2016-17	2017-18	2018-19	Total
Live Birth	5168	4440	5028	14636
Maternal Near-Miss (MNM)	64	56	33	153
Maternal Death (MD)	19	09	10	38
Maternal Mortality Ratio (MMR)	367.6	202.7	198.9	259.6
Severe maternal outcome ratio (SMOR)	16.1	14.6	8.6	13.1
MNM incidence ratio (MNMIR)	12.4	12.6	6.56	10.5
Maternal near-miss: mortality ratio (MNM: 1MD)	3.4:1	6.2:1	3.3:1	4:1
Mortality index (MI)	22.9%	13.8%	23.3%	19.9%

Cesarean section accounted for 56.2% and 57.9% of delivery in near miss and mortality cases, respectively (Table 5). In the near-miss group, 14.4% of women underwent vaginal delivery as compared to 23.7% in the mortality group.

**Table 5:** Outcome of pregnancy in maternal near-miss cases and maternal deaths

Pregnancy Outcome	Maternal Near Miss n (%) (N = 153)	Maternal Death n (%) (N = 38)
Vaginal Delivery	22 (14.4)	9 (23.7)
Cesarean Section	86 (56.2)	22 (57.9)
Laparotomy for ectopic pregnancy	20 (13.1)	0
Curettage/vacuum aspiration	15 (9.8)	1 (2.6)
Others	10 (6.5)	6 (15.8)

Hemorrhagic disorders of pregnancy were the leading cause of near-miss (40.5%), followed by hypertensive disorder (25.5%), cardiac illness (14.4%), and sepsis (7.8%). Both hemorrhage (23.7%) and sepsis (23.7%) constituted the leading causes of mortality, followed by hypertensive disorders of pregnancy (15.8%). Moreover, the post-partum hemorrhage, eclampsia, sepsis, and peripartum cardiomyopathy were the independent conditions associated with higher morbidity and mortality (Tables 6-9).

**Table 6:** Underlying disorders in maternal near-miss cases and maternal deaths

Underlying disorder	Maternal near-miss n (%) (n = 153)	Maternal death n (%) (n = 38)
Hemorrhagic disorders of pregnancy	62 (40.5)	9 (23.7)
Hypertensive disorders of pregnancy	39 (25.5)	6 (15.8)
Cardiac diseases	22 (14.4)	3 (7.9)
Sepsis or severe systemic infection	12 (7.8)	9 (23.7)
Hepatic disorder	11 (7.2)	3 (7.9)
Dengue fever	0	3 (7.9)
Other medical or surgical conditions	6 (3.9)	5 (13.2)

**Table 7:** Types of hemorrhagic disorder in maternal near-miss cases and maternal deaths

Hemorrhagic disorders	Maternal near miss n (%) (n = 62)	Maternal death n (%) (n = 9)
Post-partum hemorrhage	21 (33.9)	8 (88.9)
Antepartum hemorrhage	11 (17.7)	1 (11.1)
Ruptured uterus	9 (14.5)	0
Ruptured ectopic pregnancy	15 (24.2)	0
Incomplete abortion	6 (9.7)	0

**Table 8:** Types of hypertensive disorder in maternal near-miss cases and maternal deaths

Hypertensive disorders	Maternal Near miss n (%) (N = 39)	Maternal Death n (%) (N = 6)
Severe Preeclampsia	9 (23.1)	0
Eclampsia	28 (71.8)	6 (100)
HELPP Syndrome	2 (5.1)	0

**Table 9:** Types of cardiac disease in maternal near-miss cases and maternal deaths

Cardiac diseases	Maternal near miss n (%) (N = 22)	Maternal death n (%) (n = 3)
Valvular heart disease	8 (36.4)	0
Peripartum cardiomyopathy	9 (40.9)	3 (100)
Congenital heart disease	2 (9.1)	0
Others	3 (13.6)	0

Table 10 depicts the organ dysfunction associated with near-miss and mortality groups. On audit of these cases, we found that 62% of near-miss and 92% of mortality group patients admitted with one or more organ dysfunction on admission. Moreover, 22.9% of near-miss and 7.9% mortality group patients admitted with a disorder but without any organ dysfunction but later developed it during their stay in hospital. Only 15% of patients in the near-miss group were healthy on admission (Table 11). 

**Table 10:** Organ dysfunction in maternal near-miss cases and maternal deaths

Organ dysfunction	Maternal near miss n (%) (n = 153)	Maternal Death n (%) (n = 38)
Cardiovascular dysfunction	31 (20.3)	6 (15.8)
Respiratory dysfunction	11 (7.2)	0
Renal dysfunction	09 (5.9)	0
Coagulation/hematologic dysfunction	24 (15.7)	8 (21)
Hepatic dysfunction	14 (9.2)	2 (5.3)
Neurologic dysfunction	27 (17.6)	5 (13.2)
Uterine dysfunction/hysterectomy	15 (9.8)	0
Multiorgan Dysfunction	22 (14.4)	14 (36.8)

**Table 11:** Status of maternal near-miss and maternal death cases on admission to the hospital

On admission status of cases	Maternal near miss n (%) (n = 153)	Maternal death n (%) (n = 38)
Cases presenting with organ dysfunction on admission	95 (62.1)	35 (92.1)
Cases admitted with disorder developed organ dysfunction	35 (22.9)	3 (7.9)
Cases admitted without any disorder developed organ dysfunction	23 (15)	0

## Discussion

MMR has been used by healthcare planners as the yardstick to assess the quality of obstetric services in an area. Lately, the focus has shifted from maternal mortality to maternal near-miss as a more valuable indicator of maternal health [3].

However, there was a lack of clarity in the inclusion criteria of near-miss cases. Near-miss cases were either identified based on disease [4] or critical intervention (Hysterectomy, ICU admission, etc.) [5] or organ-dysfunction [6] using laboratory parameters with pros and cons in each criterion. Recently, the WHO expert group authorized organ system failure or dysfunction-based criteria as the standard in identifying near-miss cases [2]. In this study, the WHO near-miss criteria based on organ dysfunction or failure was used. Patients merely having an obstetric complication without any derangement in laboratory parameters or patients who needed ICU admission for postoperative monitoring without any systemic disorder or organ dysfunction were not categorized as near-miss. 

The MMR in our hospital during the study period was 259.6, which was much higher compared to the national figure of 130 [7]. That probably due to the lack of adequate health infrastructure and services in the surrounding areas, (East Singhbhum district, Jharkhand) especially in the remote rural areas. According to government data, there are only nine PHCs (primary health centre), 16 APHCs (additional public health centre), and 242 HSCs (health sub-centre) catering to a population close to 23 lakhs, which is much less than the national recommendation (a PHC for every 30,000 population) [8-9]. The MNMR (number of maternal near-miss cases per 1,000 live births) was 10.5 in our study, and the severe maternal outcome ratio (SMOR), which refers to the number of women with life-threatening conditions including death, was 13.1. Both MNMR and SMOR give an estimate of the amount of care and resources that would be needed to improve obstetric care in that area or facility. We reviewed studies done in different parts of India, and the near-miss ratio ranged from 120 to 10.4 (Table 12) [10-22]. However, all these studies have used different criteria in identifying near-miss cases and hence technically not ideal for comparison. In other subcontinent countries, the incidence of the near-miss was 5.5, 26.8, and 7.13 in studies conducted in Sri Lanka [23], Bangladesh [24], and Pakistan [25], respectively.

**Table 12:** List of studies on maternal near-miss done in different parts of India LB: live birth; MMR: maternal mortality ratio; MNMR: maternal near-miss ratio; MNMMR: maternal near-miss mortality ratio; MI: mortality index

Author	Year	Setting	Criteria for near-miss	LB	MMR	MNMR	MNMMR	MI	Near miss conditions
PS [10]	2013	Tertiary care hospital, Manipal	Disease-specific management based	7330	313	17.8	5.6: 1	14.9	Haemorrhage (44.2), hypertension (23.6), sepsis
Pandey [11]	2014	Medical college, Lucknow	Management based	5,273	4,684	120	2.6:1	-	Haemorrhage (45.7), hypertension (23.6)
Bakshi [12]	2015	Multicentre trial, Dehradun	Disease-specific	688	1453	74	5.1:1	16.4	Sepsis (58.8), hemorrhage (37.2), hypertension (23.5)
Singh [13]	2016	Tertiary care hospital, Raipur	Disease-specific management based organ dysfunction	13,895	734	15.2	2:1	32.58	Hypertension (38.8), haemorrhage (22.2)
Parmar [14]	2016	Tertiary care hospital, Vadodara	Disease-specific management based laboratory criteria	1929	933	23.8	2.6:1	28.1	-
Rathod [15]	2016	Tertiary care hospital, Yavatmal	Organ dysfunction	22,092	298.7	7.3	3.4:1	29.07	Haemorrhage (26.7), anaemia (24.8), hepatitis (16.7)
Sinha [16]	2016	Medical college, Bareilly	Management based (ICU admission)	8051	484.4	10.8	2.2:1	-	Hypertension (22.2)
Ray [17]	2016	Tertiary care hospital, Karad	Disease-specific	4038	421	54.5	13:1	7.17	Hypertension (56), haemorrhage (11)
Chandak [18]	2017	Medical college, Nagpur	Disease-specific management based organ dysfunction	12757	243	10.7	4.4:1	-	Hypertension, haemorrhage
Tallapureddy [19]	2017	Medical college, Hyderabad	Disease-specific management based	3784	158.5	8.4	5.3:1	15.79	Haemorrhage (43.7%), hypertension (31.2%)
Gupta [20]	2018	Medical college, Indore	Disease-specific management based organ dysfunction	4533	330.9	16.3	3.5:1	22.1	Haemorrhage (40.5), hypertension (24.3), sepsis (13.5)
Mansuri [21]	2019	Four tertiary care hospitals, Ahmedabad	Organ dysfunction	21,491	367.6	11.49	3.13:1	24.23	Hypertension, haemorrhage
Verma [22]	2020	Tertiary care hospital, Etawah	Disease-specific	8638	891.4	16.6	1.9:1	34.8	Hypertension (45.7), hemorrhage (23.5)

The indicators relating near miss and mortality (maternal near-miss mortality ratio and mortality index) represent a fair account of overall available health resources and the quality of health care in that area. Maternal near-miss mortality ratio (MNMMR) refers to the ratio between maternal near-miss cases and maternal deaths, and a higher ratio indicates better quality of care as more women survived than died. Similarly, the mortality index represents the percentage of the patient with life-threatening conditions who ultimately die. In this case, a higher value means more women with life-threatening conditions die, indicating the low quality of care and vice versa [2]. In our study, MNMMR was 4:1, and the mortality index was 19.9, which is comparable to most of the Indian studies.

As expected, in resource-limited countries like India, more women suffer and die as compared to the European countries, mainly due to lacunae in managing obstetric emergencies at various levels. Three delays have been identified, which are believed to be the reason for poor obstetric emergency care in the Indian setup [26]. The first delay is the lack of awareness leading to delay in availing health care facilities. The second delay is inaccessibility to health care due to lack of transport, cost, or socioeconomic issues. The third delay is related to lack of proper care at the health care facility due to the delay in diagnosis of critical conditions or decision making or unavailability of resources or trained health care provider. On analyzing this aspect in our study, we found that 92% of maternal deaths and 62% of near-miss cases were having one or more organ dysfunction on admission, which suggests that the first and the second delays could be the primary reason for the high morbidity and mortality in our study. This hypothesis is also indirectly supported by the fact that about 84% of patients of near-miss and 92% of maternal death were un-booked (no prior antenatal visit). 

Like most of the Indian studies, haemorrhage (40.5%) accounted for the leading cause of morbidity, followed by hypertensive disorders (25.5%) in our study. Conversely, both sepsis and haemorrhage were responsible for almost half of the cases of maternal death. Post-partum haemorrhage and eclampsia constituted the major complications among hemorrhagic disorder and in the hypertensive disease group, respectively. Peripartum cardiomyopathy (40.9%) was the leading cause of cardiac morbidity in our study, and it was the only cause of cardiac-related maternal mortality. That was unlike in most parts of India, where rheumatic heart disease and congenital heart diseases were the leading cause of morbidity and mortality [27-28]. It may be because most of the valvular heart diseases were diagnosed early in the pregnancy and hence optimized adequately before delivery, whereas cardiomyopathies are usually diagnosed late. 

A number of initiatives have been undertaken in our hospital to improve our response to obstetric emergencies over the last few years. The numbers of staff in the labour room have been augmented. In-house 24-hour anaesthesia service has started. An obstetric high dependency unit (HDU) has been established. Fast-tracking of blood and blood product availability from the blood bank has been streamlined. We also introduced a weekly analysis of near-miss and mortality cases to identify preventable factors and implementing protocols adhering to National Guidelines and current evidenced-based practices. In addition to that, the training of caregivers, labour room nurses, and midwives at regular intervals were strengthened. However, the impact of these quality initiatives on obstetric care needs to be quantitatively assessed by a future study.

Looking at the health care deficiencies found in our study area, there is a need to improve community-level resources and most importantly, awareness regarding antenatal visits among the public. Health care providers working in primary health centres (PHC) and community health centres (CHC) may need training in managing maternal complications, and the government should take initiatives in strengthening the referral system of sick patients to higher centres.

## Conclusions

The current study was conducted to assess the incidence and causes of maternal near-miss and mortality in a tertiary care hospital situated in eastern India. The near-miss cases were identified by using the WHO criteria of near-miss. The study revealed that the near-miss cases had similar pathological courses as that of maternal mortality. Haemorrhage, hypertensive disorders, and sepsis were the major reasons for morbidity and mortality like in other parts of India. It was also observed that most of the near-miss and mortality cases didn’t have a prior antenatal visit. Moreover, most of them were severely ill with pre-existing organ dysfunction on admission to the hospital, suggesting a delay in seeking proper care or delay in reaching the health-care facility. A limitation of this study was that it involves only the patients coming to a single tertiary care hospital. Hence, extrapolating the data of this study to the whole population of this area may be erroneous. A further study may be warranted to evaluate the root causes of the health care gaps at the community level. To conclude, maternal near-miss can be compared with the black box of an aircraft as they survived from similar complications to that of maternal mortality and they are alive to provide useful information regarding the inadequacies in maternal health care.
